# Identification of Host Genes Involved in Geminivirus Infection Using a Reverse Genetics Approach

**DOI:** 10.1371/journal.pone.0022383

**Published:** 2011-07-26

**Authors:** Rosa Lozano-Durán, Tábata Rosas-Díaz, Ana P. Luna, Eduardo R. Bejarano

**Affiliations:** Málaga-Consejo Superior de Investigaciones Científicas (IHSM-UMA-CSIC), Departamento Biología Celular, Genética y Fisiología, Universidad de Málaga, Campus Teatinos, Málaga, Spain; Instituto de Biología Molecular y Celular de Plantas, Spain

## Abstract

Geminiviruses, like all viruses, rely on the host cell machinery to establish a successful infection, but the identity and function of these required host proteins remain largely unknown. *Tomato yellow leaf curl Sardinia virus* (TYLCSV), a monopartite geminivirus, is one of the causal agents of the devastating Tomato yellow leaf curl disease (TYLCD). The transgenic 2IRGFP *N. benthamiana* plants, used in combination with Virus Induced Gene Silencing (VIGS), entail an important potential as a tool in reverse genetics studies to identify host factors involved in TYLCSV infection. Using these transgenic plants, we have made an accurate description of the evolution of TYLCSV replication in the host in both space and time. Moreover, we have determined that TYLCSV and *Tobacco rattle virus* (TRV) do not dramatically influence each other when co-infected in *N. benthamiana*, what makes the use of TRV-induced gene silencing in combination with TYLCSV for reverse genetic studies feasible. Finally, we have tested the effect of silencing candidate host genes on TYLCSV infection, identifying eighteen genes potentially involved in this process, fifteen of which had never been implicated in geminiviral infections before. Seven of the analyzed genes have a potential anti-viral effect, whereas the expression of the other eleven is required for a full infection. Interestingly, almost half of the genes altering TYLCSV infection play a role in postranslational modifications. Therefore, our results provide new insights into the molecular mechanisms underlying geminivirus infections, and at the same time reveal the 2IRGFP/VIGS system as a powerful tool for functional reverse genetics studies.

## Introduction

Geminiviruses are a large family of plant viruses with circular, single stranded DNA genomes packaged within geminate particles [1]. The *Geminiviridae* family [2] is divided into four genera according to their genome organization and biological properties. The genus *Begomovirus* includes members that are transmitted by whiteflies, infect dicotyledonous plants, and may have either bipartite or monopartite genomes. *Tomato yellow leaf curl Sardinia virus* (TYLCSV) is a member of the *Begomovirus* genus, and is one of the causal agents of the Tomato yellow leaf curl disease (TYLCD), which can cause up to 100% yield losses in tomato fields [3], [4], [5]. TYLCSV has a monopartite genome of 2.8 kb in size, which encodes six proteins and contains an intergenic region (IR) comprising the origin of replication and viral promoters. The open reading frames (ORFs) in the complementary sense orientation encode a replication-associated protein (Rep, also known as C1), a transcriptional activator protein (TrAP, also known as C2), and a replication enhancer protein (REn, also known as C3); a small ORF, C4, is located within the Rep ORF but in a different reading frame. The virion strand contains two ORFs encoding the coat protein (CP) and a small protein named V2 [4], [5].

To establish a successful infection, viruses must create a proper environment for viral propagation, which involves hijacking the cellular machinery for viral functions and, at the same time, preventing or counteracting the plant defence mechanisms. To fulfil these requirements, viral proteins trigger changes in the cell at all levels: transcriptional, translational and posttranslational. Identifying the host genes involved in viral replication, movement, and generally all those processes that lead to the establishment of a successful infection, could provide valuable new targets to ultimately generate viral resistance.

The advances in high-throughput technologies and bioinformatics have made possible to assess gene expression massively, providing an insight into the host's transcriptional responses to viral infections in a genome-wide fashion. These transcriptomic studies, together with proteomic studies, are providing an unprecedented vision of the “host-side” of the plant-virus interaction, leading to the identification of host genes whose function or expression is altered as a consequence of the infection. Geminiviruses have also been recently the subject of this kind of study, unveiling host genes differentially expressed either during the infection [6], [7], [8] or upon expression of a viral protein [8], [9], [10]. However, despite all this information being available, it is still a daunting task to determine the exact role of these host genes in the infection process. Notably, this is particularly challenging in the case of monopartite geminiviruses, in which gene replacement with marker genes is not feasible, and thus are more tedious to monitor. In a previous work, we described the generation of *Nicotiana benthamiana* transgenic plants containing a GFP (Green fluorescence protein) expression cassette flanked by two repeats of TYLCSV IR as a tool to monitor TYLCSV replication [11]. These plants, named 2IRGFP, entail an important potential as a tool in reverse genetics studies to identify host factors involved in the viral infection, when used in combination with VIGS (Virus Induced Gene Silencing) technology. Although the feasibility of this approach was previously confirmed by silencing the Proliferating cellular nuclear antigen (PCNA) and Sumo conjugating enzyme (SCE1) genes [11], [12], its use in a larger screening required an optimization of the conditions.

In this work, we explore further the potential of 2IRGFP *N. benthamiana* plants in combination with VIGS to identify host genes with a role in geminivirus infection. We have achieved an accurate description of the dynamics of viral replication by monitoring GFP expression in both space and time, explored the limitations of the strategy to be used in a reverse-genetics screening, and unveiled the effect of silencing selected *N. benthamiana* genes, most of them previously identified in transcriptomic or protein-protein interaction studies, in geminivirus infection. Using this strategy, we have identified eighteen genes involved in several cellular processes whose silencing alters TYLCSV infection. Notably, for fifteen of these genes this is the first description of a role in viral infections. Hence, our results provide new insights into the molecular mechanisms underlying geminivirus infections, and at the same time reveal the 2IRGFP/VIGS system as a powerful tool for functional reverse genetics studies.

## Results

### Dynamics of *Tomato yellow leaf curl Sardinia virus* infection in transgenic 2IRGFP *N. benthamiana* plants is not altered by co-infection with *Tobacco rattle virus*

Traditionally, the development of geminivirus infections has been monitored by symptom evaluation and quantification of viral DNA by nucleic acid hybridization or PCR [13], [14]. These methods, however, have important limitations to monitor the infection in both space and time. Symptom evaluation is semi-quantitative at best, and does not necessarily correlate with viral accumulation. Hybridization or PCR studies, on the other hand, are destructive methods that are not able to discriminate if the viral molecules accumulated in a certain plant organ or tissue have been produced *in situ* or, on the contrary, have been originated elsewhere and subsequently transported. Due to these restrictions, a comprehensive study of the dynamics of the geminivirus infection, considering active replication and not merely virus accumulation, is still lacking.

In a previous work [11], we developed *N. benthamiana* transgenic plants that overexpress GFP in those cells where the virus is replicating. During TYLCSV infection, these plants, named 2IRGFP, display a Rep-dependent GFP overexpression driven by the generation of mGFP replicons. Since overproduction of GFP correlates with TYLCSV active replication, these plants provide an unprecedented opportunity to monitor TYLCSV infection. For this purpose, 2IRGFP plants were infected with TYLCSV (three independent experiments, 20 plants each), GFP expression was exhaustively monitored and samples were collected at different times post-infection. For each time point, three plants were sampled (one per independent experiment); for each of the sampled plants, the three most apical leaves were taken, and tissue printing was performed with the main root. Total DNA was extracted from the harvested leaves, and both mGFP replicons and viral DNA were detected by DNA hybridization.

**Figure 1:**
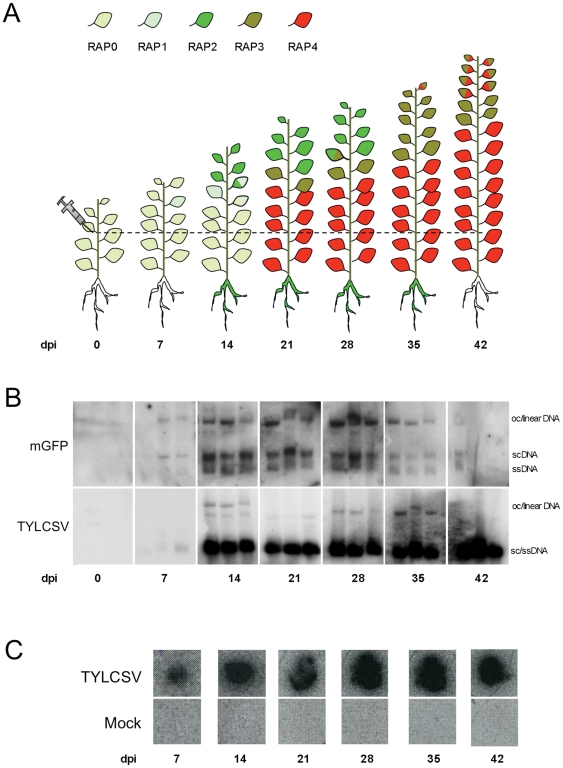
Phenotypic and molecular analysis of TYLCSV-infected 2IRGFP *N. benthamiana* plants. (A) Evolution of RAP phenotypes in TYLCSV-infected transgenic *N. benthamiana* 2IRGFP plants. The diagram displays the average RAP phenotypes of leaves and the induction of GFP in roots at different days post-infection (dpi). Leaves containing areas of two different colours indicate an equivalent coexistence of RAP phenotypes in the population. In roots, green colour indicates GFP overexpression. The depicted results are the average of 60 infected plants. The dashed line marks the inoculation point. (B) Detection of episomal replicons (mGFP) and virus (TYLCSV) in leaves of infected plants. DNA was extracted from the three most apical leaves of three independent plants infected with TYLCSV. Undigested DNA was blotted and hybridized with probes specific for mGFP or TYLCSV. Bands representing open circle (oc), supercoiled (sc) or single-stranded (ss) forms of DNA are indicated. (C) Detection of virus (TYLCSV) in roots of infected plants in tissue printing.

According to the extension and intensity of GFP expression in leaves, we visually distinguished five phenotypes, which we named RAP (for Replication-Associated Phenotype) 0, 1, 2, 3 and 4, as depicted in Figure S1. Leaves from uninfected plants show a low expression of GFP extended through the whole leaf surface (RAP0). In RAP1, which corresponds to the first stage of the virus infection, GFP overexpression appears in some of the vascular bundles and the background GFP expression is not extensively silenced. RAP2 represents the stage of maximum GFP accumulation, in which an intense green fluorescence is observed as a continuous pattern through the leaf vascular bundles, and the GFP expression background in the leaf lamina has faded. RAP3 is the last stage in GFP expression, where GFP can only be detected in distinct areas of the leaf vascular bundles, before it completely disappears (RAP4). The average evolution of GFP expression in the leaves of TYLCSV-infected plants is depicted in Figure 1A. At 7 days post-infection (dpi), GFP over-expression associated to RAP1 phenotype can already be observed in some, but not most, plants, and accumulation of mGFP replicons and viral DNA is already detectable (Figure 1B). One week later, at 14 dpi, the maximum levels of viral replication, monitored as GFP overexpression (RAP2), are reached in the most apical leaves. As expected, this increase in GFP correlates with a higher accumulation of mGFP replicons and viral DNA. The RAP2 phenotype is maintained in the apical leaves up to 28 dpi, while GFP silencing is extensively detected from 21 dpi in the rest of the leaves. The decrease in GFP over-expression observed from 35 dpi onwards (Figure 1A) correlates to the reduction of mGFP replicons (Figure 1B); the viral accumulation, however, is high, most likely due to previous rounds of replication. As seen in this figure, TYLCSV is also replicating in the roots between 14 and 35 dpi, as indicated by GFP overexpression. The appearance of GFP in roots correlated with presence of viral DNA in the tissue printing (Figure 1C) until 42 dpi, when no GFP can be observed but accumulation of viral DNA is detected. This viral DNA is most likely the result of previous viral replication in the root, or even in the aerial parts of the plant. It is noteworthy that viral DNA could be detected in roots as early as 7 dpi, before GFP expression is clearly noticeable; bearing in mind that the root is a sink organ, this is probably the result of transport from leaves where the virus is actively replicating (Figure 1B).

Once an extensive description of the dynamics of TYLCSV infection has been achieved, detecting changes in the timing or pattern of GFP over-expression due to silencing of a given host gene should be easy and reliable. *Tobacco rattle virus* (TRV)-based silencing vectors have been widely used and offer several advantages over other viral vectors, such as their abilities to mediate VIGS in the absence of TRV-derived symptoms and to target host RNAs in the growing points of plants. To accurately evaluate the impact of TRV infection on the evolution of the RAP phenotype, we monitored the GFP expression in 2IRGFP plants co-infected with TRV and TYLCSV (three independent experiments, 20 plants each). TRV/TYLCSV co-infected plants showed the same pattern of RAP phenotypes described for TYLCSV infected plants; the only detectable difference between single and double infected plants is a slight delay of approximately two days in the appearance of RAP phenotypes.

### TYLCSV infection does not revert TRV-induced gene silencing in *N. benthamiana*

**Figure 2:**
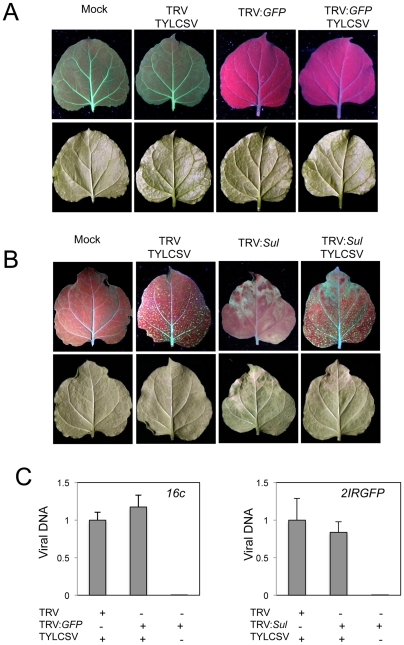
Effect of TYLCSV infection on TRV-induced silencing of *GFP* or *Sul.* Leaves from *N. benthamiana* 16c (A) or 2IRGFP (B) transgenic *N. benthamiana* plants 15 days after inoculation with TRV:*GFP* or TRV:*Sul*, respectively, or co-inoculation with TRV or TRV:*GFP*/*Sul* and TYLCSV. (C) Relative amount of TYLCSV DNA determined by quantitative real-time PCR. Values are the mean of five replicates. Bars represent standard error.

Since several proteins encoded by TYLCSV can function as suppressors of gene silencing (A. P. Luna et al., in preparation), TYLCSV infection might interfere with the TRV-induced silencing. To test this possibility, we evaluated the effect of TYLCSV infection on the silencing of either a *GFP* transgene or the endogenous *Sulfur* (*Sul*) gene. To determine the impact of TYLCSV infection on the silencing of the *GFP* transgene, *N. benthamiana* plants constitutively expressing GFP (line 16c) [15], [16] were co-infected with TRV:*GFP* and TYLCSV or infected with TRV:*GFP* alone as a control. Infection with TRV:*GFP* triggered the silencing of the transgene, and this silencing was fully extended by 15 dpi (Figure 2A). Co-infection with TYLCSV did not alter this silencing phenotype, indicating that TYLCSV does not interfere with the TRV-induced *GFP* silencing (Figure 2A).

The *Sul* gene was chosen to evaluate the effect of TYLCSV infection on the silencing of an endogenous gene, for it produces a readily visible phenotype when silenced, derived from its involvement in chlorophyll synthesis [17]. 2IRGFP *N. benthamiana* plants were co-infected with TRV:*Sul* and TYLCSV or infected with TRV:*Sul* alone as a control. Once again, co-infection with TYLCSV did not affect the silencing phenotype of TRV:*Sul* infected plants (Figure 2B), indicating that TYLCSV does not alter the TRV-induced silencing of this endogenous gene.

Quantification of TYLCSV accumulation using quantitative real-time PCR shows that TRV-induced silencing of either *GFP* or *Sul* does not affect TYLCSV accumulation (Figure 2C).

### Simultaneous TRV-induced silencing of multiple genes in *N. benthamiana* plants

One drawback to VIGS is that it very often does not produce a uniform silencing throughout the plant. If the silencing of the gene does not generate a readily visible phenotype, it will be very difficult to distinguish silenced from non-silenced tissues, what would dramatically complicate the interpretation of results. A strategy to compensate for the lack of uniformity of VIGS would incorporate an internal reference to monitor the level of silencing. This system would act as a control for the VIGS vector, marking the silenced areas with a visible phenotype. The use of internal markers for VIGS based in visual phenotypes has been implemented in several plant species and has proven very successful for empowering the method as a tool in reverse genetics. Some works have demonstrated that the simultaneous silencing of several genes is possible by including multiple gene sequences in the same silencing vector [18], [19], [20]. With the aim of developing a visual reporter system to mark silenced areas in *N. benthamiana* leaves, we decided to follow two different approaches: (*i*) Test if the silencing triggered by two distinct TRV constructs co-localize, and (*ii*) Test if the silencing triggered by two different gene sequences cloned in tandem in the same TRV vector co-localize. For these assays we used two gene sequences whose silencing produces a readily visible phenotype: the *Sul* gene and *PCNA* [11], [21].

The results obtained are presented in Figure S2. In our system, silencing of the two marker genes does not significantly co-localize when the two TRV clones are co-inoculated in the plant. Only 13.6% of the new leaves in co-inoculated plants displayed both phenotypes, and the percentages of leaves showing each phenotype considered separately are lower than in single inoculations, indicating that co-inoculation apparently leads to a decreased silencing efficiency. A similar effect is observed when both genes are cloned in tandem in the same TRV vector, although the percentage of leaves showing simultaneous *Sul-* and *PCNA*-silenced phenotypes is slightly higher (20%) (Figure S2). Segregation of the silencing phenotypes warns against the use of this strategy as a marker system for gene silencing in *N. benthamiana* leaves.

### Selection and cloning of candidate genes

As a first step in the identification of host genes required for TYLCSV infection, we made a selection of candidate genes following several criteria: (*i*) Genes encoding proteins known to physically interact with geminivirus proteins; (*ii*) Genes exclusively or preferentially expressed in phloematic tissues; (*iii*) Genes transactivated by the C2 homologue from the geminiviruses *Mungbean yellow mosaic virus* and *African cassava mosaic geminivirus*; (*iv*) Genes involved in cellular processes potentially required for geminivirus infection (Table 1). A total of 114 genes were initially included as candidate genes.

**Table 1:** List of candidate genes.

Identity	Function	Selection criteria	Reference	ACC *A. thaliana*
**Group A (no detected effect on infection)**				
A-type cyclin-dependent kinase (*CDK2*)	Cell cycle control	Cellular process	[6]	AT3G48750
Cullin-associated and neddylation-dissociated (*CAND1*)	Protein metabolism	TrAP/C2 interaction	Hericourt *et al*. (in preparation)	AT2G02560
DNA polymerase alpha 2 (*POLA2*)	DNA metabolism	Cellular process	[80]	AT1G67630
DNA polymerase delta small subunit (*POLD2*)	DNA metabolism	Cellular process	[80]	AT2G42120
E2F transcription factor 1 (*E2FB*)	Transcription	Cellular process	[6]	AT5G22220
Geminivirus Rep-interacting kinase (*GRIK1*)	Signal transduction	Rep interaction	[81]	AT3G45240
Histone 3 K4-specific methyltransferase SET7/9	Unknown	TrAP/C2 interaction	Hericourt *et al*. (in preparation)	AT1G21920
Homologue to co-chaperone DNAJ-like protein (*ATJ3*)	Protein folding	C3 interaction	Hericourt *et al*. (in preparation)	AT3G44110
NSP interacting kinase 2 (*NIK2*)	Signal transduction	Phloem over-expression	[82]	AT3G25560
Putative nucleic acid binding/transcription factor (*JDK*)	Unknown	TrAP/C2 interaction	Hericourt *et al*. (in preparation)	AT5G03150
Putative transcriptional activators with NAC domain (*ATAF1*)	Transcription	C3 interaction	[83]	AT1G01720
Putative shikimate kinase (*SKL2*)	Unknown	CP interaction	Hericourt *et al*. (in preparation)	AT2G35500
Retinoblastoma-related protein (*RBR*)	Cell cycle control	Rep interaction	[84], [85]	AT3G12280
RUB-activating enzyme subunit (*ECR1*)	Protein modification	Cellular process	[6], [86]	AT5G19180
Scarecrow-like protein (*SCL13*)	Transcription	Phloem over-expression	[82]	AT4G17230
SNF1-related protein kinase (*AKIN11*)	Signal transduction	TrAP/C2 interaction	[87]	AT3G29160
SUMO activating enzyme (*SAE1B*)	Protein metabolism	Cellular process	[88]	AT5G50580
Transcription factor IIA gamma chain (*TFIIA-S*)	Transcription	Phloem over-expression	[89]	AT4G24440
Wound inducive gene (*F14P1.1*)	Stress	C4 interaction	Hericourt *et al*. (in preparation)	AT1G19660
**Group B (promote earlier infection)**				
Bearskin 2 (*BRN2*)	Transcription	Phloem over-expression	[89]	AT4G10350
Importin alpha isoform 4 (*IMPA-4*)	Transport	CP interaction	[71]	AT1G09270
Lactoylglutathione lyase (*GLO1*)	Stress	C3 Interaction	Hericourt *et al*. (in preparation)	AT1G15380
Replication protein A32 (*RPA32/RPA2*)	DNA metabolism	Rep interaction	[32]	AT3G02920
Dehydration responsive 21 (*RD21*)	Stress	V2 interaction	Hericourt *et al*. (in preparation)	AT1G47128
RING-type E3 ubiquitin ligase (*RHF2A*)	Protein modification	Transactived by TrAP/C2	[82]	AT5G22000
Ubiquitin activating enzyme (*UBA1*)	Protein modification	TrAP/C2 Interaction	Hericourt *et al*. (in preparation)	AT2G30110
**Group C (delay, reduce or prevent the infection)**				
4-coumarate:CoA ligase (*AT4CL1*)	Metabolism	Phloem over-expression	[89]	AT1G51680
Allene oxide cyclase (*AOC1*)	Metabolism	Phloem over-expression	[82]	AT3G25760
Barely any meristem 1 *(BAM1)*	Protein modification	C4 interaction	Hericourt *et al*. (in preparation)	AT5G65700
Coatomer delta subunit (*deltaCOP*)	Protein transport	C3 interaction	Hericourt *et al*. (in preparation)	AT5G05010
COP9 signalosome subunit 3 (*CSN3*)	Protein modification	Cellular process	[90]	AT5G14250
Geminivirus Rep A-binding (*GRAB2*)	Transcription	Rep interaction	[29]	AT5G61430
Heat shock protein cognate 70 (*HSC70*)	Protein modification	Phloem over-expression	[82]	AT5G02500
Nuclear acetyltransferase (*NSI*)	Signal transduction	NSP Interaction	[31]	AT1G32070
Patatin-like protein 2 (*PLP2*)	Stress	Phloem over-expression	[82]	AT2G26560
Shaggy-related kinase kappa (*SK4-1/SKK)*	Protein modification	C4 interaction	Hericourt *et al*. (in preparation)	AT1G09840
SKP1-like 2 (*ASK2*)	Protein modification	Transactived by TrAP/C2	[9]	AT5G08590

**Figure 3:**
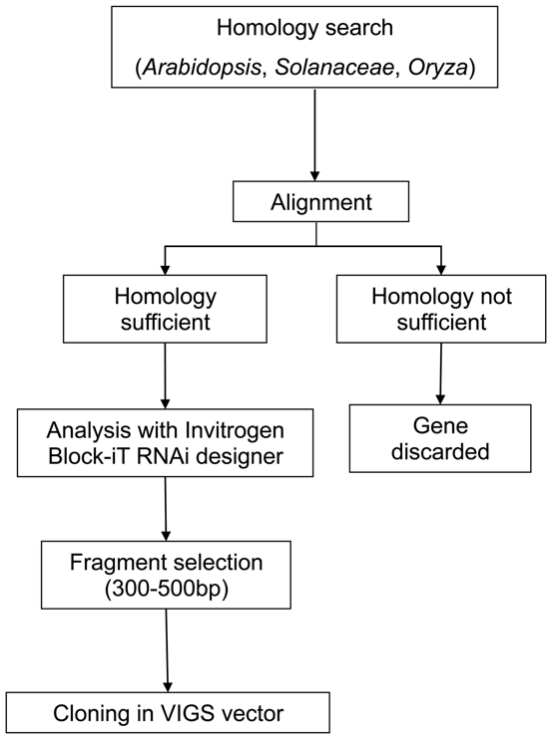
Gene selection strategy. Flow diagram depicting the strategy used for selecting the candidate genes to be tested using the 2IRGFP plants/TRV-based system.

The criterion for selection is indicated in each case. The accession numbers (ACC) of the homologous *Arabidopsis* gene used in the VIGS experiments are indicated in this case.

silencing could be reached by expressing a DNA fragment of 21 to 23 nucleotides bearing 100% identity to the target gene [22], this is often not efficient at triggering silencing and longer sequences must be used [22], [23]. The highest efficiency of VIGS appears to be achieved using fragments in the range of 300–500 nucleotides with multiple stretches of more than 23 nucleotides identity [24], [25]. Because of their different sources, our candidate genes belong to different species. Cloning 300–500 bp fragments of the *N. benthamiana* homologous gene would be the strategy of choice; unfortunately, the *N. benthamiana* genome has not been sequenced yet and thus the gene sequences are in most cases not available. To circumvent this difficulty, we carried out homology analyses in all selected genes to identify sequences of 300–500 bp conserved in different plant species, including *Arabidopsis* and tomato. The use of heterologous gene sequences to silence their respective orthologs in *N. benthamiana* has been previously reported [26]. Chosen sequences were further analysed with Invitrogen Block-iT™ RNAi designer (https://rnaidesigner.invitrogen.com/rnaiexpress/) to localize potential efficient siRNAs within the sequence: the fragment of choice was that containing the largest number of proposed siRNA molecules. The selection process is depicted in the flow diagram in Figure 3.

After this analysis, 54 out of the initial 114 genes were maintained as candidate genes (Table 1). Since the sequence of these selected genes was highly conserved, we decided to use the *Arabidopsis* cDNAs to generate the VIGS constructs, with the aim of rendering this strategy faster and more homogeneous. We ordered the 42 *Arabidopsis* cDNA clones that were available at NASC (European *Arabidopsis* Stock Centre) (Table S1) and the selected 300–500 bp fragment for each cDNA was PCR-amplified and cloned in the TRV RNA2-based VIGS vector pTV00 [27]. The primers used to amplify each fragment are included in Table S1.

### Screening of candidate genes in *N. benthamiana* 2IRGFP plants

**Figure 4:**
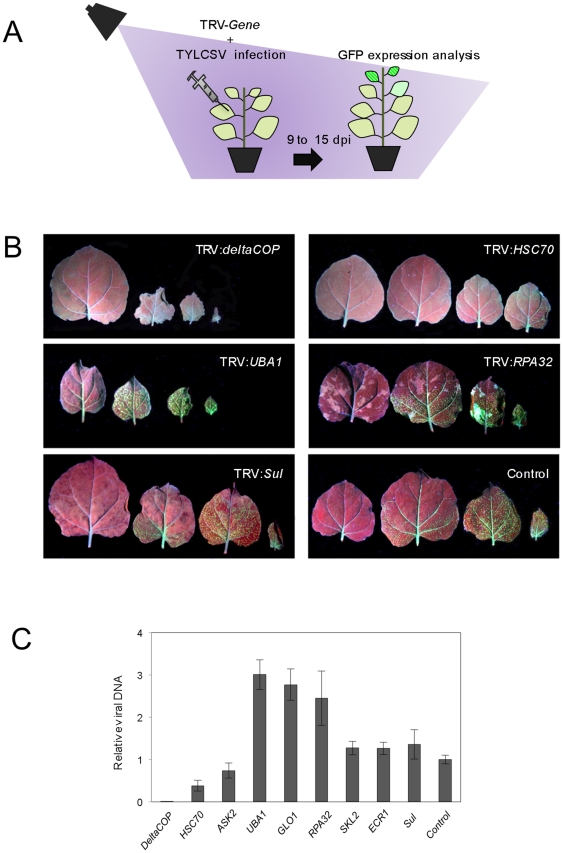
Screening of candidate genes in 2IRGFP transgenic *N. benthamiana* plants. (A) Plants were co-inoculated with a TRV:*Gene* construct and TYLCSV. GFP expression was monitored daily from 9 to 15 dpi. Five plants were used per construct; experiments were repeated at least twice. (B) GFP expression in the four most apical leaves of 2IRGFP transgenic plants co-infected with TYLCSV and representative TRV constructs. (C) Relative amount of TYLCSV DNA in leaves of plants co-infected with TYLCSV and TRV constructs to induced the silencing of either Coatomer delta subunit (*deltaCOP*), Heat shock cognate 70 (*HSC70*), SKP1-like 2 (*ASK2*), Ubiquitin activating enzyme 1 (*UBA1*), Lactoylglutathione lyase (*GLO1*), Putative shikimate kinase (*SKL2*), RUB-activating enzyme subunit (*ECR1*), Replication associated protein A (*RPA32*), *Sulfur* (*Sul*) or no gene (empty vector, as control). Viral DNA was quantified by quantitative real-time PCR. Values are the mean of five replicates. Bars represent standard error. The sample of TYLCSV and pTV00 co-infected plants was used as the calibrator, with the expression level of the TYLCSV capsid protein gene set to 1.

Once the time course of TYLCSV infection in 2IRGFP plants had been established, we followed the strategy depicted in Figure 4A to test the potential effect of candidate gene silencing on TYLCSV infection (Table 1). Summing up, we induced gene silencing for each candidate host gene in 2IRGFP plants using TRV constructs, and subsequently infected these plants with TYLCSV. Plants infiltrated with the empty TRV vector and infected with TYLCSV were used as a control; plants infiltrated with the *Sul*-containing TRV vector were used as a control of VIGS efficiency. GFP overexpression was monitored daily from 9 to 15 dpi under UV light.

According to the effect of their silencing on TYLCSV infection, measured as time of appearance and intensity of GFP expression, we grouped the tested host genes into three classes: those whose silencing did not cause changes in GFP expression (group A), or those whose silencing promoted earlier (group B) or later/lower/null (group C) GFP expression (Table 1; examples of each class are shown in Figure 4B).

Representative genes belonging to groups A (*SKL2*, *ECR1*), B (*UBA1*, *GLO1* and *RPA32*) and C (*HSC70*, *ASK2*, and *deltaCOP*) were chosen to evaluate the impact of their silencing on TYLCSV infection, measured as viral DNA accumulation. For this purpose, 2IRGFP *N. benthamiana* plants were co-inoculated with the TRV derivative clones and TYLCSV. At 15 dpi, total DNA was extracted from the pooled three most apical leaves of each plant and the relative amount of viral DNA was determined using quantitative real-time PCR (two independent experiments, 5 plants each**)**. The mean values of TYLCSV accumulation are represented in Figure 4C. As expected from the GFP overexpression data, silencing of *UBA1* or *GLO1* and silencing of *RPA32* tripled and doubled TYLCSV accumulation, respectively. On the other hand, silencing of *HSC70* and *ASK2* reduced TYLCSV accumulation by 70 and 30%, respectively. Strikingly, silencing of the *deltaCOP* subunit completely abolished TYLCSV accumulation.

## Discussion

### Replication dynamics of TYLCSV

Transgenic 2IRGFP *N. benthamiana* plants have proven to be an accurate and sensitive tool that allows monitoring TYLCSV infection real-time and in a non-destructive manner. Using these transgenic plants, we have been able to describe the dynamics of TYLCSV infection in great detail, determining in which tissues the virus is replicating on an average infection at a certain time. To our knowledge, this is the first description of the replication dynamics of a geminivirus infection in both space and time, as most of the previous studies reflect viral DNA accumulation but not active replication.

According to our results, TYLCSV replication can be detected in leaves placed above the inoculation point at 7 dpi. One week later (14 dpi), viral replication is taking place in the apical leaves of all inoculated plants, where it is maintained at a high level until 28 dpi. From that moment onwards, the rate of viral replication decreases, and eight weeks after the inoculation it is only detectable in limited areas of apical leaves. These observations suggest that the virus is able to maintain the replication of its genome, in the aerial parts of the host plant, only in certain leaves and during a limited period of time. Additionally, the virus is also able to replicate in roots between 14 and 35 dpi. Interestingly, while we observe a direct correlation between the changes in GFP expression and the accumulation of episomal replicons (mGFP), the amount of viral DNA seems to be maintained even when viral replication can no longer be detected. These data suggest that, although both DNA molecules are produced by the same mechanism, mGFP replicons must be degraded whereas the viral DNA is not, maybe as a result of its encapsidation.

### Double infection with TYLCSV and TRV does not significantly affect TYLCSV infection or TRV-induced silencing

We have demonstrated that co-infection with TRV does not dramatically affect TYLCSV infection in *N. benthamiana*. This fact makes it feasible to use TYLCSV in combination with TRV-mediated VIGS as a tool in reverse genetics studies to identify host factors involved in the geminivirus infection. We observed, however, a slight delay in the development of TYLCSV infection when in combination with TRV. This delay makes the use of appropriate controls (co-infection with the empty TRV vector) of special importance for this type of analysis. Although TYLCSV, like all geminiviruses, encodes suppressors of gene silencing (A. P. Luna et al., in preparation), it does not noticeably affect TRV-induced gene silencing in *N. benthamiana* plants. In agreement with these results, TRV-mediated VIGS has been successfully used in combination with geminiviral infections in tomato in a recent work [28].

Despite our efforts, the attempt to establish a visual reporter system based on the silencing of the *Sulfur* gene has been fruitless. Although simultaneous silencing of two genes is achieved by both co-infiltration of independent TRV-based constructs or by infiltration with a TRV construct harbouring multiple gene sequences (Figure S2), silencing does not significantly co-localize in any case, and the extension of the silencing of each gene considered independently diminishes (Figure S2). Even though the reasons for this outcome remain obscure, the absence of significant co-localization makes it impractical to use this co-silencing approach as a marker for VIGS. A similar effect of simultaneous silencing in *N. benthamiana* had been previously described [21].

### Identification of host genes involved in TYLCSV infection

Using our reverse genetics approach, based on the use of transgenic 2IRGFP *N. benthamiana* plants, we have been able to demonstrate that silencing of 18 out of 37 analysed host genes alters TYLCSV infection.

Bearing in mind the limitations of VIGS, and since we have not tested the silencing of those candidate genes in which no effect on TYLCSV infection could be detected (group A), we cannot rule out the possibility that we may have false negatives: some of the tested genes might not have been efficiently silenced, and thus their potential impact on the viral infection would go unnoticed. For this reason, we cannot assess that those tested candidate genes without an obvious effect on TYLCSV infection do not play a role in the viral infection. False positive results, on the other hand, would be more difficult to obtain in our experimental system, and as long as the proper controls are being used we consider the positive results as reliable. In this context, a reasonable concern would be the possibility of silencing unwanted host genes as a consequence of sequence homology with the target host gene. In order to evaluate this undesired effect, we performed a BLAST homology search with every sequence used for VIGS, confirming that the only hit in each case was the selected target gene. However, and since the *N. benthamiana* genome has not been sequenced yet, this is a possible risk that should be kept in mind.

Additionally, it is noteworthy that this screening method tests the candidate gene in the context of the infection, and consequently those genes identified should be biologically relevant.

Out of the eighteen genes whose silencing alters TYLCSV infection, seven have a potential anti-viral effect, since TYLCSV replication is enhanced when they are silenced (group B), whereas the expression of the other eleven is required for a full infection, for their silencing negatively impacts this process (group C).

Among the genes affecting TYLCSV infection, there are three (*NSI*, *GRAB2* and *RPA32*) whose deregulation was previously shown to modify the geminivirus infection or replication [29], [30], [31], [32].

An earlier work showed that overexpression of the nuclear acetyltransferase NSI, a protein that interacts with the Nuclear shuttle protein (NSP) of the geminivirus *Cabbage leaf curl virus* (CaLCuV), enhances the efficiency of infection [30], suggesting a role for protein acetylation in coordinating replication of the viral genome with its export from the nucleus. This positive effect of NSI in the geminivirus infection is supported by the data obtained with TYLCSV, which demonstrate that silencing of NSI negatively affects viral infection. On the other hand, silencing of the Geminivirus RepA binding gene (*GRAB2*) during TYLCSV infection has an opposite effect on viral propagation to that previously reported for a different geminivirus species [29]. This gene encodes a NAC-containing protein isolated in wheat for its interaction with *Wheat dwarf virus* (WDV) RepA [29]. Even though GRAB2 overexpression inhibits WDV replication in wheat cells, the reason for this remains unclear, and could be ascribed to different roles of GRAB2 on the viral DNA cycle [29]. Our results show that reduction in gene expression of GRAB2 has a deleterious effect on TYLCSV infection, suggesting that correct GRAB2 expression is required for full infectivity. Replication Protein A (RPA32) has been shown to interact with *Mungbean yellow mosaic India virus* (MYMIV) Rep [32] and modulate the functions of Rep by enhancing its ATPase, but down-regulating its nicking and closing activities. Strikingly, even though RPA32 seems to promote the transient replication of a plasmid bearing MYMIV origin of replication *in planta* [32], in our system its silencing seems to enhance the viral infection. We do not have a feasible explanation for this contradictory phenotype at the moment, and further work will be needed to decipher it.

The roles of other host genes whose silencing affects TYLCSV infection might be deduced from their known cellular functions. Therefore, we will briefly discuss below the potential roles of a group of identified host factors with known cellular functions in postranslational modifications, stress responses, metabolism or intracellular transport.

It is noteworthy that 8 out of these 18 genes are involved in processes related to protein modifications or protein metabolism, such as ubiquitination, rubylation, phosphorylation, acetylation or protein folding.

Four of these genes encode components or regulators of the ubiquitin or ubiquitin-like pathways: Ubiquitin activating enzyme (*UBA1)*, RING-type E3 ubiquitin ligase (*RHF2A*), SKP1-like 2 (*ASK2*) and a subunit of the de-rubylating CSN complex (*CSN3*).

Ubiquitination has been shown to contribute to multiple levels of plant defence, including resistance to viruses (reviewed in [33] and [34]). Specifically, several recent works have suggested the existence of links between ubiquitination and geminivirus infection [6], [10], [35], [36]. Since the tomato UBA1 interacts with TYLCSV C2 (F. Hèricourt et al., in preparation), the finding that silencing of this host gene leads to an earlier TYLCSV infection suggests that the interaction with the viral C2 protein might lead to the inhibition of the enzyme, which would be consistent with the previously described general negative impact of C2 on the ubiquitination in the host [10]. On the other hand, the expression of the RING-type E3 ubiquitin ligase *RFH2A* silenced in this work is up-regulated following CaLCuV infection [6] or infiltration with virulent *Pseudomonas syringae* (*Arabidopsis* eFP browser: http://esc4037-shemp.csb.utoronto.ca/efp/cgi-bin/efpWeb.cgi), which may indicate an involvement in plant defence. Such a hypothetical role would explain why the silencing of this gene promotes the viral infection.

The SCF complex seems to be an important target during geminivirus infection, since several geminiviral proteins interfere with or hijack the SCF function [10], [37]. The fact that three of the genes whose silencing alters TYLSCV infection are components or regulators of these complexes supports this idea. *ASK2* is a member of a gene family encoding SKP1-like proteins that can be assembled into distinct SCF complexes, and plays a role in a large number of cellular processes such as cell division, development, osmotic stress or drought tolerance [38], [39], [40]. *ASK2* expression is down-regulated by challenge with bacteria, fungi or elicitors (*Arabidopsis* eFP browser) but transactivated by geminivirus C2 in *Arabidopsis* protoplasts [9], suggesting a possible involvement in plant defence acting as a negative regulator. If this is the case, it could explain the adverse effect of its silencing on TYLCSV infection.

CSN3 is one of the eight subunits of the CSN complex, which derubylates cullins and thus regulates the activity of ubiquitin Cullin RING Ligases (CRLs). Recently, geminivirus C2 protein was shown to interfere with the activity of this complex over CULLIN1, most likely through the interaction with CSN5, the catalytic subunit, therefore altering ubiquitination in the host cell [10]. Given that geminivirus infection on *Arabidopsis csn5a* mutant plants takes place less efficiently that in wild-type plants (Lozano-Durán and Bejarano, submitted), it might be feasible that geminiviruses could be redirecting the activity of the CSN complex, rather that generally impairing it. Since depletion of any of the CSN subunits results in the loss of the complex (reviewed in [41]), it would not be surprising that silencing of CSN3 results in a hindered infection.

Among the host genes that seem to be required for the viral infection, since their silencing delay or suppress TYLCSV replication, we identified two encoding protein kinases that interact with TYLCSV C4 (Héricourt et al., in preparation): *BAM1* (Barely any meristem 1) and *SK4-1/SKK* (Shaggy-related kinase kappa). *BAM1* encodes a CLAVATA1-related receptor kinase-like protein required for both shoot and flower meristem function, which is also involved in leaf and gametophyte development [42], [43], [44]. Interestingly, *BAM1* expression is down-regulated after challenge with fungi, bacteria or elicitors (*Arabidopsis* eFP browser). In such a scenario, silencing of this gene might lead to an activation of defence responses in the plant. Alternatively, since this protein interacts with TYLCSV C4 (Héricourt et al., in preparation), this gene product might be required for some viral function.

Shaggy-like protein kinases like *SK4-1/SKK* have been shown to interact with other geminiviral C4 proteins, and this interaction is required to trigger disease symptoms [45], [46] and for C4 function to suppress gene silencing [46]. Our results confirm the previous idea that these kinases might be required for geminivirus infection, since silencing of *SK41/SKK* negatively impacts TYLCSV infection.

Five of the identified genes potentially involved in TYLCSV infection have a role in stress responses: *HSC70-1* (Heat shock protein cognate 70), *RD21* (responsive to dehydration 21), *PLP2* (patatin-like protein), *GLO1* (lactoylglutatione lyase) and *AOC1* (allene oxide cyclase 1). HSC70-1 is one of the five cytosolic members of the heat shock protein 70 family in *Arabidopsis* [47]. Infection with several plant viruses, such as the geminivirus *Beet curly top virus*, induce the expression of members of this gene family in systemically infected tissues [48], [49] HSC70 is a major interactor of SGT1 [50], which has proven required for resistance to viruses [51], [52]. A chloroplastic HSC70 from *Arabidopsis*, CPHSC70-1 (At4g24280), has been recently shown to interact with *Abutilon mosaic virus* movement protein, and this interaction seems to be important for viral transport and symptom induction [53]. Although the role of HSC70 induction in plant-virus interaction is uncertain, it might be expected to fulfil a requirement for rapid protein maturation and turnover during a short virus multiplication cycle. Alternatively, there is evidence that HSC70 may play a role in virus cell-to-cell movement. Our results show that silencing of HSC70-1 results in an impaired TYLCSV infection, supporting that over-production of this protein is required for a full viral infection.

RD21 is a cysteine protease whose homologue in tomato is able to interact with TYLCSV V2 (F. Héricourt et al., in preparation). RD21 has been recently shown to be the target protease of the serpin AtSerpin1 [54]. In animals, serpins are protease inhibitors involved in several physiological processes, including innate immunity. The expression of *RD21* is up-regulated following inoculation with *Botrytis cinerea* or *Pseudomonas syringae (Arabidopsis* eFP browser*)*, or upon CaLCuV infection [6], suggesting a possible role of RD21 in plant defence, which would in turn explain why the silencing of this gene promotes the viral infection.

*PLP2* encodes a lipid acyl hydrolase that accumulates upon infection with CaLCuV [6], fungi and bacteria and negatively affects resistance to the last two types of pathogens [55]. On the contrary, it has been shown to contribute to resistance to *Cucumber mosaic virus* by inducing HR [56]. Since this gene product is proposed to positively regulate the biosynthesis of oxylipins providing fatty acid precursors [56], silencing of this gene might result in increased salicylic acid signalling, which could explain the impairment of TYLCSV infection.

*GLO1* is part of the glyoxalase system, involved in detoxification of methylglioxal (MG), a cytotoxic byproduct of glycolysis (reviewed in [57]). Overexpression of the glyoxalase pathway in transgenic tobacco and rice plants has been found to keep in check the increase of ROS and MG under stress conditions by maintaining glutathione homeostasis and antioxidant enzyme levels (reviewed in [57]), and overexpression of *GLO1* has been related to enhanced tolerance to abiotic stresses [58], [59]. A possible role for reactive oxygen species as a requirement for virus replication [60] and for antioxidative mechanisms as antagonizing viral infection [59] has been proposed. Moreover, viral infections have been shown to induce oxidative stress in plants [61], [62], [63], [64], [65], [66] and geminivirus infection alters the expression of oxidative stress-related genes [6]. Given that silencing of *GLO1* triggers an earlier TYLCSV infection, it would be feasible that its interaction with C3 might be interfering with this enzyme to promote pathogenicity.

*AOC1* is one of four genes that encode this enzyme in *Arabidopsis*, which catalyzes an essential step in jasmonic acid biosynthesis. This gene is repressed upon CaLCuV infection [6], maybe as a consequence of the opposite regulation between jasmonate and salicylic acid signalling pathways, since the latter is activated in this geminivirus-host interaction. Due to this counter-regulation, silencing of this gene might result in activation of the salicylic acid pathway in response to TYLCSV, explaining its negative effect on the viral infection.

Viruses heavily rely on cytoplasmic transport systems for their propagation. Among the host factors involved in TYLCSV infection, we have identified one gene required for vesicular trafficking (Coatomer delta subunit, *deltaCOP*) and another one involved in transport between the cytoplasm and the nucleus (Importin alpha isoform 4, *IMPAA-4*).

*deltaCOP* encodes a component of the polymeric coatomer coat complexes COPI. The precise role of the COPI remains unclear, although it has been associated with vesicular transport within the Golgi apparatus and from the Golgi apparatus to the ER [67]. Vesicular trafficking has been previously shown to play a role in geminivirus infection, since interaction with synaptotagmin SYTA has proven required for CaLCuV cell-to-cell movement and systemic spread [68]. Interestingly, silencing of this gene completely abolishes TYLCSV infection in our system, suggesting that vesicular trafficking in essential for viral infection.

IMPAA-4 is one of the members of the importin α gene family in eukaryotes. Importin α is a component of the nuclear pore-targeting complex (PTAC) that acts as an adaptor by recognizing the nuclear localization signal (NLS) sequences and binding to importin β. Importin β is the carrier component of PTAC, and targets the complex to the nuclear pore by binding to nuclear pore proteins [69], [70]. Importin α has been shown to interact with the CP from the geminivirus MYMV [71], and this interaction might serve for docking of viruses to the nucleus and facilitating nuclear localization of the CP during encapsidation. In this context, the finding that silencing of *IMPA-4* favours the viral infection seems counterintuitive; however, the fact that this gene is overexpressed in response to several pathogens and elicitors (*Arabidopsis* eFP browser) suggests that this host factor might also play a role in plant defence, providing a possible explanation for the observed phenotype. Additionally, TYLCSV CP could rely on the interaction with a different host protein for its nuclear import.

Besides the aforementioned cellular processes, others seem to be involved in TYLCSV infection. Silencing of genes selected because of their specific expression or overexpression in phloem tissue and required for phenylpropanoid metabolism (4-coumarate:CoA ligase1, *4CL1*) or secondary cell wall synthesis (Bearskin2B, *BRN2*) delay or promote TYLCSV infection, respectively. BRN2 is a member of the Class IIB NAC transcription factor family. In *Arabidopsis*, this protein has been suggested to regulate cell maturation in cells that undergo terminal differentiation with strong cell wall modifications [72]. *4CL1* is involved in the last step of the general phenylpropanoid pathway, channeling carbon flow into branch pathways of the phenylpropanoid metabolism. Interestingly, silencing of this gene leads to increased cellulose content and reduced amounts of total lignin [73].

As illustrated in the examples above, the use of this approach has allowed the identification of novel plant genes with a role in the geminivirus infection, which sheds light on the underlying biological processes, therefore paving the way for the development of strategies to counteract these devastating diseases. Given the previously mentioned advantages of this 2IRGFP/VIGS system, it can be considered an easy, fast and effective tool to determine the role of host genes in geminivirus infections, and might be of great assistance to speed up this kind of functional studies. However, using VIGS to target a specific gene requires information about its nucleotide sequence. This is a limitation when working with *N. benthamiana,* as there is only a relatively small sequence database available for this species (http://www.tigr.org). We have tried to circumvent this difficulty by using nucleotide sequence information from *Arabidopsis* and closely related species. In a genome era, full sequencing of the *N. benthamiana* genome should hopefully be fulfilled in the near future, providing full potential to the VIGS/2IRGFP strategy to identify host factors involved in geminivirus infection.

## Materials and Methods

### Microorganisms, plants and general methods

Manipulations of *Escherichia coli* and nucleic acids were performed according to standard methods [74], [75]. *E. coli* strain DH5-α was used for subcloning. All PCR-amplified fragments cloned in this work were fully sequenced. *Agrobacterium tumefaciens* GV3101 strain was used for the delivery of *Tobacco rattle virus* (TRV) RNA2-based vectors and TYLCSV infective clone; *A. tumefaciens* C58c1 was used for the delivery of the TRV RNA1-based construct pBINTRA6 [27].

2IRGFP *N. benthamiana* plants were grown in soil at 22°C in short day conditions (8 h light/16 h dark photoperiod).

### Plasmids and cloning

cDNA clones of the selected candidate genes were obtained from the *Arabidopsis* Information Resource (TAIR) (Table S1). Fragments (300–500 bp) from the selected genes were generated by PCR with specific primers (Table S1) and cloned in pGEMT-easy (Promega). *Spe*I/*Apa*I fragments from the pGEMT clones containing the selected sequenced were subcloned into *Spe*I/*Apa*I sites of TRV RNA2-based vector pTV00 [27] to yield the correspondent TRV used to silencing the plants genes.

To yield the TRV:*GFP* construct, a 383 bp *BamH*I-*Cla*I fragment from pSMGFP [76] was cloned into *BamH*I-*Cla*I of pTV00. To yield the TRV:*Sul* construct, a 450 bp fragment of the *Sulfur* gene amplified from *Arabidopsis* cDNA using At*Sulfur* primers (Table S1) was digested with *Kpn*I and cloned into the *Kpn*I site of pTV00. To yield the TRV:*SulPCNA* construct a 450 bp *Kpn*I fragment from TRV:*Sul* was subcloned into *KpnI* site of TRV:*PCNA* [11].

### Geminivirus infection assays and detection of viral and mGFP DNA

Viral infections of 2IRGFP *N. benthamiana* plants were performed by the agroinoculation technique as previously described [77]. Plants were agroinoculated with plasmid pGreenTYA14 (binary vector containing a partial dimer of TYLCSV-ES[2] [10]) in the axilary bud of the fourth/fifth leaf of 3-week-old wild-type or transgenic 2IRGFP *N. benthamiana* plants. For control, plants were mock inoculated with *A. tumefaciens* culture harbouring the empty binary vector pGreen-0229 [78].

Viral and mGFP DNAs were detected by gel blot hybridization. Total plant DNA was extracted from *N. benthamiana* leaves at different days postinfection. Two micrograms of undigested total DNA per sample were used. As probe for TYLCSV detection, we used a *BamH*I DNA fragment from pGreenTYA14 [10] containing a full-length genome of TYLCSV-ES. For mGFP detection we used a *BamH*I-*Sac*I DNA fragment from pSMGFP comprising the complete GFP open reading frame [76].

For quantitative real-time PCR, total plant DNA was extracted from *N. benthamiana* leaves at 15 dpi. The reaction mixture consisted of approximately 10 ng total DNA, primer mix (3 µM each) and SYBR Green Master Mix (TaKaRa, Kyoto, Japan) in a total volume of 25 µl. The PCR conditions were: 10 minutes at 95°C, and 40 cycles of 30 seconds at 95°C and 30 seconds at 60°C. The reactions were performed using a Rotor-Gene real time cycler (QIAGEN, Hamburg Germany). A relative quantification real-time PCR method using the 2^ΔΔCT^ method [79] was used to compare the amount of the TYLCSV capsid protein gene (amplified using primers GGAGGCTGAACTTCGACAGC and GGACTTTCAATGGGCCTTCAC) between different infections/experiments. The 25S ribosomal DNA interspacer (ITS) (amplified using primers ATAACCGCATCAGGTCTCCA and CCGAAGTTACGGATCCATTT) was used as the internal control.

### Virus Induced Gene Silencing assay

Virus induced gene silencing with TRV in *N. benthamiana* plants were performed according the method described by [27]. Briefly, independent cultures of *A. tumefaciens* GV3101 carrying pTV00 or pTV00-based constructs and *A. tumefaciens* C58c1 carrying pBINTRA6 were grown overnight in LBroth medium plus appropriate antibiotics. Cultures were resuspended in VIGS buffer (10 mM morpholineethanesulfonic acid pH 5.6, 10 mM MgCl_2_, and 100 µM acetosyringone) adjusting optical density to OD_600_ = 1, and incubated overnight at room temperature in the dark. Cultures containing pBINTRA6 plasmid and pTV00 or pTV00-derived plasmid were mixed at a 1∶1 ratio. Approximately 1 mL of this mixed culture was used to infiltrate the underside of two leaves of each 3-week-old 2IRGFP *N. benthamiana* plant.

## Supporting Information

Figure S1 **Phenotypes of TYLCSV-infected 2IRGFP** ***N. benthamiana*** **plants.** Extension and intensity of GFP expression in the leaves of TYLCSV-infected plants corresponding to RAP phenotypes (for Replication-Associated Phenotype) 0, 1, 2, 3 and 4.
(TIF)

Figure S2 **Simultaneous TRV-induced silencing of** ***PCNA*** **and** ***Sul.*** Percentage of leaves located above the infiltration point displaying the silencing phenotype of either *PCNA, Sul* or both in *N. benthamiana* plants inoculated with TRV:*Sul*, TRV:*PCNA* or TRV:*SulPCNA*, or co-inoculated with TRV:*Sul* and TRV:*PCNA.* For each inoculation, n = 10 plants. The data correspond to leaves collected approximately 28 days after the infection.
(TIF)

Table S1 **Oligonucleotides used for amplifying and cloning fragments of the selected genes.**
(DOC)
