# Supplementary figures and images for: Identification of Host Genes Involved in Geminivirus Infection Using a Reverse Genetics Approach
Source: PLoS One. 2011 Jul 26;6(7):e22383. doi: 10.1371/journal.pone.0022383 (PMC3144222; doi:10.1371/journal.pone.0022383)

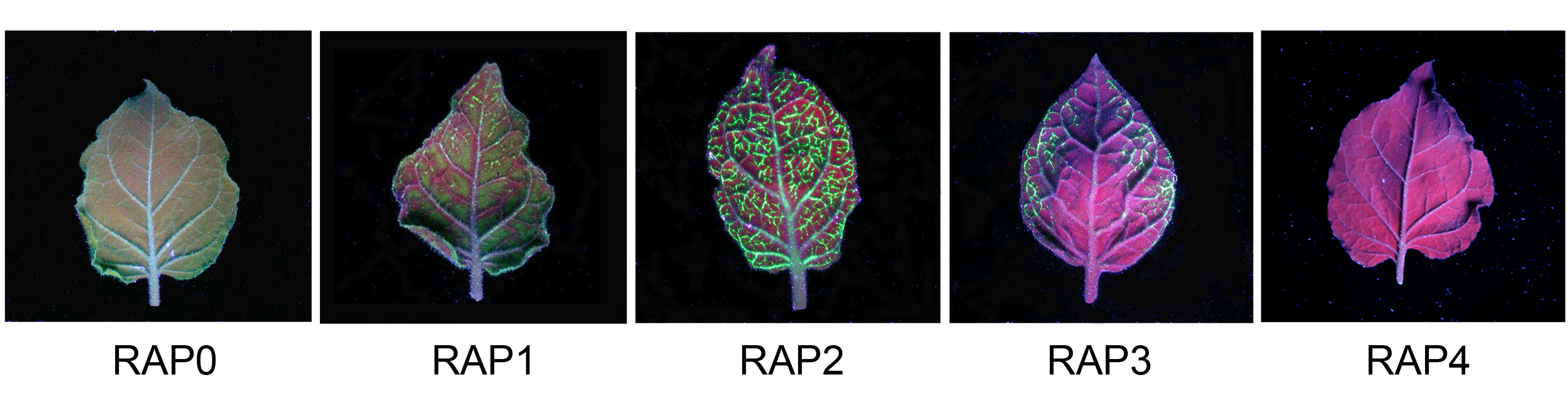

Supplement: Figure S1 — Phenotypes of TYLCSV-infected 2IRGFP N. benthamiana plants. Extension and intensity of GFP expression in the leaves of TYLCSV-infected plants corresponding to RAP phenotypes (for Replication-Associated Phenotype) 0, 1, 2, 3 and 4. (TIF) [file pone.0022383.s001.tif]

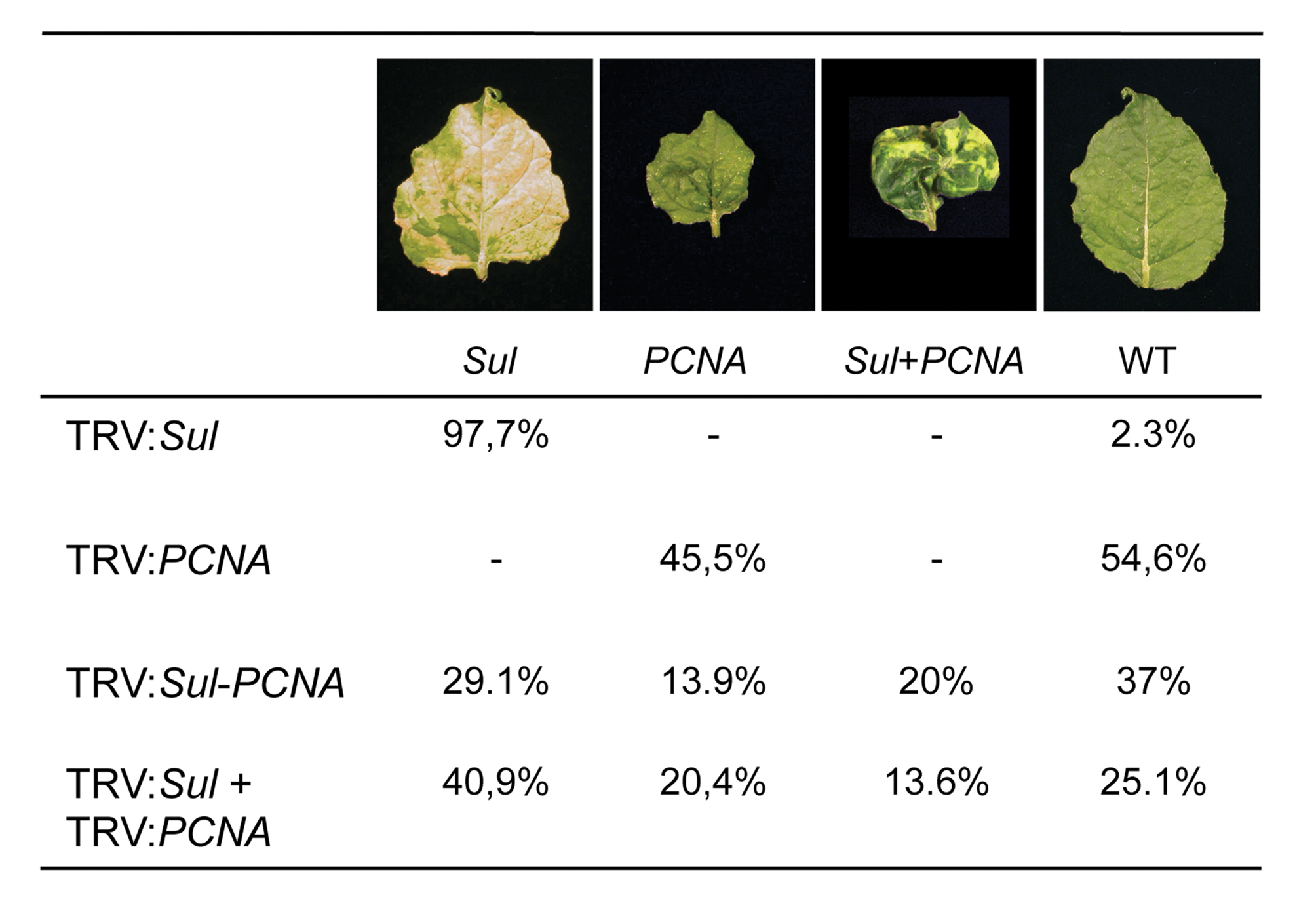

Supplement: Figure S2 — Simultaneous TRV-induced silencing of PCNA and Sul. Percentage of leaves located above the infiltration point displaying the silencing phenotype of either PCNA, Sul or both in N. benthamiana plants inoculated with TRV:Sul, TRV:PCNA or TRV:SulPCNA, or co-inoculated with TRV:Sul and TRV:PCNA. For each inoculation, n = 10 plants. The data correspond to leaves collected approximately 28 days after the infection. (TIF) [file pone.0022383.s002.tif]
