# Supplementary material for: Identification of Host Genes Involved in Geminivirus Infection Using a Reverse Genetics Approach
Source: PLoS One. 2011 Jul 26;6(7):e22383. doi: 10.1371/journal.pone.0022383 (PMC3144222; doi:10.1371/journal.pone.0022383)
Supplement: Table S1 — Oligonucleotides used for amplifying and cloning fragments of the selected genes. (DOC) [file pone.0022383.s003.doc]

**Table S1.** Oligonucleotides used for amplifying and cloning fragments of the selected genes.

| **Gene** | **TAIR clone** | **Forward** | **Reverse** |
| --- | --- | --- | --- |
| A-type cyclin-dependent kinase (*CDK2*) | U83549 | ATATGATCAAAACATATC | CCCATGATTCTGAAAATC |
| Allene oxide cyclase (*AOC1*) | U82314 | AAGTTCAAGAACTGAGCG | AACCTGTCCGTAGGCACC |
| At*Sulfur* (*Sul*) | - | AACCGAGGTACCGTTTGCCC | CAGAAGGTACCAAAGCCTTTGAGC |
| Barely any meristem 1 (*BAM1*) | U25612 | TCGGTGAGGTGCTTCACG | TCCGACAGGTTTTCTTCC |
| Bearskin 2 (*BRN2*) | [C103095](http://www.arabidopsis.org/servlets/TairObject?id=938547&type=clone) | ACCAAAAGTTTGAGATGG | TCTACATACCACCCATCC |
| Coatomer delta subunit *(deltaCOP/*) | [U16159](http://www.arabidopsis.org/servlets/TairObject?id=922963&type=clone) | TCTTGTTACAACTAAGC | TTCAAGTCTTCCAGATCC |
| COP9 signalosome subunit 3 (*CSN3*) | [U14097](http://www.arabidopsis.org/servlets/TairObject?id=723575&type=clone) | CAGGTGTTAATTTCTCTAC | CAAACGCTTGGAGGAGACCTG |
| Cullin-associated and neddylation-dissociated (*CAND1*) | U25698 | TATTGGTTCACTTGTTCC | GATAGATTAATGTGAAGC |
| Dehydration responsive 21 (*RD21*) | [U11707](http://www.arabidopsis.org/servlets/TairObject?id=938661&type=clone) | AACTTACAGCGAGGAATCG | AGGCTTGATGGGAGATGG |
| DNA polymerase alpha 2 (*POLA2*) | [U61004](http://www.arabidopsis.org/servlets/TairObject?id=1001140145&type=clone) | CAGATTTCATCAATCCCG | TCTGAATATTACATTTGC |
| DNA polymerase delta small subunit (*POLD2*) | [U24549](http://www.arabidopsis.org/servlets/TairObject?id=1001230334&type=clone) | TCTTCACCTTATAACACC | TAGGAATGCAGATCAACC |
| E2F transcription factor 1 (*E2F1*) | [U25624](http://www.arabidopsis.org/servlets/TairObject?id=1001228839&type=clone) | CTTAAGAACAGGATTCAG | GTCTATTGGTCCCATTG |
| Geminivirus Rep A-binding (*GRAB2*) | [U15787](http://www.arabidopsis.org/servlets/TairObject?id=721807&type=clone) | AGAGCAGATGGATTTACC | AGAATTTTCCTTCAAGCC |
| Geminivirus Rep-interacting kinase (*GRIK1*) | [U09417](http://www.arabidopsis.org/servlets/TairObject?id=635911&type=clone) | GCTAAGTCGTTCCAAACG | ACCTCAATGAGATTAACG |
| Heat shock protein cognate 70 (*HSC70*) | [U09493](http://www.arabidopsis.org/servlets/TairObject?id=721090&type=clone) | ATTGACTCTCTATACGAG | ACCTTAGGGATACGGGTAG |
| Histone 3 K4-specific methyltransferase SET7/9 | [U09782](http://www.arabidopsis.org/servlets/TairObject?id=500939675&type=clone) | GAGAGTTGGGCTAGAGG | CTTGCTTACGACCTTCATGC |
| Homologue to co-chaperone DNAJ-like protein (*ATJ3*) | [C104790](http://www.arabidopsis.org/servlets/TairObject?id=500939822&type=clone) | CTCTTGAGGATGTGTACC | TCCAGTGACAGTGTCAGG |
| 4-coumarate:CoA ligase (*AT4CL1*) | [U21582](http://www.arabidopsis.org/servlets/TairObject?id=1000939625&type=clone) | CGAATTCGCCACTAAGCC | GTCGACGTAACGAGCTTC |
| Lactoylglutathione lyase (*GLO1*) | [U17691](http://www.arabidopsis.org/servlets/TairObject?id=923291&type=clone) | ATCTCACGTCACTGAACC | CCTTCTTCTACTAATGCC |
| NSP interacting kinase 2 (*NIK2*) | U21612 | TGACGATTACTTTGAAGC | CTATTCTATCGTAGTTGC |
| Nuclear acetyltransferase (*NSI*) | [U82318](http://www.arabidopsis.org/servlets/TairObject?id=1002545915&type=clone) | GCTTATTGGTATGGCACG | GAAAGAACAGTTTTATCC |
| Importin alpha isoform 4 (*IMPA-4*) | [U18321](http://www.arabidopsis.org/servlets/TairObject?id=922937&type=clone) | TTTATTCTGATGATCCTC | TGAGAACAAGATTCCTGC |
| Patatin-like protein 2 (*PLP2*) | [U16519](http://www.arabidopsis.org/servlets/TairObject?type=stock&id=102032) | CTACTTTGATGTAATAGC | ATAGTAGGTTGAAGATGC |
| Putative nucleic acid binding/transcription factor (*JDK*) | PYAT5G03150 | GGCCACAACCTTCCATGG | TAATGCGTCGCAGAACGC |
| Putative shikimate kinase (*SKL2*) | [U13498](http://www.arabidopsis.org/servlets/TairObject?id=933705&type=clone) | TATCCACCAGCACTATCG | GATTTCAGTTGAATCTCC |
| Putative transcriptional activators with NAC domain (*ATAF1*) | [U82383](http://www.arabidopsis.org/servlets/TairObject?id=1002545879&type=clone) | TCAGAATTATTACAGTTGC | CGTGCATGATCCAATTGG |
| Replication protein A32 (*RPA32/RPA2*) | U82360 | ACGGCAACGCTGCTTTCG | CACAGATCGTTTCCCTTG |
| Retinoblastoma-related protein (*RBR*) | [C104790](http://www.arabidopsis.org/servlets/TairObject?id=500939822&type=clone) | GGTTCCATAGATGCATGC | GAGATGGCATTGGTTCAGC |
| RING-type E3 ubiquitin ligase (*RHF2A*) | C105154 | AGGGGCATTTGACTTCGG | TTCGTTCTTCAATCTCAGC |
| RUB-activating enzyme (*ECR1*) | [U13340](http://www.arabidopsis.org/servlets/TairObject?id=938917&type=clone) | AAGATTGTGTCTGCATGC | GCTCTTTGTCCAAACACG |
| Scarecrow-like protein (*SCL13*) | [U14082](http://www.arabidopsis.org/servlets/TairObject?id=1000939262&type=clone) | AGATCTTGGAAGCAATAG | ATATGGGAAGTTCACAAC |
| Shaggy-related kinase kappa (*SK4-1/SKK*) | [U16839](http://www.arabidopsis.org/servlets/TairObject?id=1000939300&type=clone) | ATCAGAACATGTTGTTGG | ATCACGGTGACAAAGACC |
| SKP1-like 2 (*ASK2*) | [U82510](http://www.arabidopsis.org/servlets/TairObject?id=1002733812&type=clone) | ATCCGGTTCAAGGAGGTG | CGTGCTTGCCATCATATTC |
| SNF1-related protein kinase (*AKIN11*) | [U21346](http://www.arabidopsis.org/servlets/TairObject?id=500939394&type=clone) | CATCCTCATATTATTCGG | AATTTACCTGATATAACC |
| SUMO activating enzyme (*SAE1B*) | [U18656](http://www.arabidopsis.org/servlets/TairObject?id=922831&type=clone) | GTGTTGATTTTTTCGAGAAG | ACGTCCTTCAGTTTCCTC |
| Transcription factor IIA gamma chain (*TFIIA-S*) | [U20073](http://www.arabidopsis.org/servlets/TairObject?id=723210&type=clone) | GGGGAAGTAGAGAATGGC | GCATCTCACTGTGTGAGC |
| Ubiquitin activating enzyme (*UBA1*) | U21814 | GCAAGAACTCAACAATGC | ACTTCAGAGAAAACAACC |
| Wound inducive gene (*F14P1.1*) | [U10100](http://www.arabidopsis.org/servlets/TairObject?id=636105&type=clone) | TTAACTCTAGTGTAGTGG | AGGCATTACCCACCTTTG |
